# Präklinische Ersteinschätzung am Einsatzort

**DOI:** 10.1007/s00103-022-03582-3

**Published:** 2022-09-20

**Authors:** Bonaventura Schmid, Florian Sauer, Hans-Jörg Busch

**Affiliations:** 1 Zentrum für Notfall- und Rettungsmedizin, Universitätsklinikum Freiburg, Medizinische Fakultät, Albert-Ludwigs-Universität Freiburg, Freiburg, Deutschland; 2 Sir-Hans-A.-Krebs-Str., 79106 Freiburg im Breisgau, Deutschland

**Keywords:** Sichtung, Notfallversorgung, Triage, Notfallpatient, Patientensteuerung, Initial assessment, Emergency care, Triage, Emergency patient, Patient management

## Abstract

Die Einschätzung des Zustandes von NotfallpatientInnen durch den Rettungsdienst, sei es durch NotfallsanitäterInnen oder auch NotärztInnen, ist ein essenzieller Teil der Arbeit des Rettungsdienstes. Sie dient der adäquaten Notfallversorgung vor Ort, der Indikationsstellung für eine Einweisung und Auswahl des weiterversorgenden Krankenhauses, aber auch der richtigen und schonenden Nutzung stationärer Ressourcen und somit der Gesamtheit sowie auch den individuellen NotfallpatientInnen.

Die Grundlage für eine korrekte Einschätzung bilden eine fundierte Ausbildung in präklinischer Notfallmedizin, klinische Erfahrung, aber auch entsprechende Scores und Instrumente zur Beurteilung der PatientInnen für ein einheitliches und an die aktuellen Qualitätsstandards angelehntes Vorgehen.

Die präklinische Ersteinschätzung ist sowohl entscheidend für unmittelbar lebensbedrohlich erkrankte PatientInnen als auch für PatientInnen mit weniger schwerwiegenden Erkrankungen. Das Ausmaß und die Dringlichkeit der Behandlung unterscheiden sich aufgrund der Schwere der Erkrankung oder Verletzung und müssen durch das Team des Rettungsdienstes richtig abgeschätzt werden.

## Abstract (en)

Emergency personnel assess their patients as an essential part of their daily work in emergency settings. The clinical assessment can help differentiate between immediately life-threatening conditions and milder diseases in patients. This assessment is the basis for an adequate preclinical treatment and for a rational referral to the hospital providing further care. Carried out in a thoughtful manner it allows reasonable allocation of resources for inpatient care.

Well-founded training, clinical experience, scores, and tools in the form of acronyms for assessing the patient lead to successful assessments according to quality standards.

The preclinical initial assessment is crucial for ensuring appropriate care for patients on site with life-threatening conditions as well as those with minor problems. Prioritization of a treatment depends on the severity of the condition or injury.

## Einleitung

Das Einsatzaufkommen des Rettungsdienstes verzeichnete in den letzten Jahrzehnten in Deutschland einen jährlichen Zuwachs von 5 % [1]. Parallel dazu hat sich der Aufgabenschwerpunkt des Rettungsdienstes und des Notarztdienstes über die Zeit gewandelt. Während früher die Behandlung lebensbedrohlicher Erkrankungszustände oder Verletzungen im Vordergrund standen, erstreckt sich nun das Aufgabenfeld der NotärztInnen zusehends auf akutmedizinische bis hin zu allgemeinmedizinischen Krankheitsbildern und Problemen [2]. Dies stellt sowohl das ärztliche als auch nichtärztliche Personal vor neue Herausforderungen bezüglich diagnostischer und therapeutischer Fertigkeiten. Zudem fungiert der Rettungsdienst als Gatekeeper, Vermittler und Logistiker zwischen den Sektoren des Gesundheitswesens, um den weiteren Weg der PatientInnen sinnvoll zu bahnen.

## Ausbildung und Weiterbildung

Vital bedrohliche Notfälle sind erfreulicherweise selten und machen nur einen geringen Anteil der Einsätze des Rettungsdienstes aus. So ist etwa die präklinische Notfallnarkose nur in 3–5 % aller Notarzteinsätze durchzuführen [3]. Geburtshilfliche Notfälle sind mit 0,2 % der Notarzteinsätze eine noch größere Rarität [4]. Die kardiopulmonale Reanimation wird im Schnitt nur alle 1,5 bis 3 Monate von einer/einem einzelnen NotärztIn durchgeführt [5]. Bedenkt man die schwerwiegenden Konsequenzen, müssen diese Szenarien nichtsdestotrotz sicher beherrscht werden. Durch eine alleinige Tätigkeit als Arzt/Ärztin im Rettungsdienst ist es hier schwierig eine ausreichende Qualifikation zu erwerben oder eine sichere Routine aufrechtzuerhalten [5].

Idealerweise sollte das notärztliche Personal den klinischen Versorgungsbereich der NotfallpatientInnen kennen. Es sollte aber auch ausreichend Erfahrung in der Versorgung und Einschätzung von weniger schwerwiegend erkrankten oder verletzten PatientInnen bestehen.

Dies kann nur durch eine regelmäßige klinische Arbeit gegeben sein, sollte aber durch ergänzende Aus- und Fortbildungen gestärkt werden. Fähigkeiten sowohl in der unmittelbaren Versorgung als auch in der Kommunikation mit den Betroffenen, aber auch mit dem Team sollten in Simulationen erlernt und verbessert werden.

Diese Kombination aus Erfahrung, fortgesetzter täglicher Patientenversorgung und Training von speziellen Situationen ist essenziell, um eine qualitativ hochwertige und angepasste Versorgung im Notfall gewährleisten zu können.

### Weiterbildung und Erwerb der Zusatzbezeichnung Notfallmedizin

Die Voraussetzungen zur Teilnahme am Rettungsdienst für ärztliches Personal sind deutschlandweit sehr heterogen geregelt. Als Mindeststandard wurde durch die Bundesärztekammer 2003 die Zusatzbezeichnung „Notfallmedizin“ eingeführt [6].

Der Erwerb der Zusatzbezeichnung fällt in die Zuständigkeiten der Landesärztekammern und ist bundesweit nicht einheitlich geregelt. Es werden eine 24-monatige Weiterbildung in einem Gebiet der akuten Patientenversorgung sowie – je nach Ärztekammer – 6 Monate Weiterbildung in der Anästhesie, der Intensivmedizin oder der Notaufnahme gefordert. Weiterhin ist die Absolvierung eines 80-Stunden-Kurses Notfallmedizin obligat verbunden mit der Absolvierung von 50 Einsatzfahrten als PraktikantIn auf einem arztbesetzten Rettungsmittel. Abgeschlossen wird die Weiterbildung Notfallmedizin in der Regel mit einer mündlichen Prüfung bei der Ärztekammer [7, 8]. Der Erwerb dieser Zusatzbezeichnung berechtigt zur notärztlichen Tätigkeit, es bedarf keiner erneuten Auffrischung der Kenntnisse.

Gerade vor dem Hintergrund, dass sich das Aufgabenspektrum der NotärztInnen hin zu einem akut- bis allgemeinmedizinischen Schwerpunkt wandelt, ist eine breite Erfahrung bei den notärztlichen KollegInnen jenseits der unmittelbar lebensrettenden Fähigkeiten von enormer Bedeutung. Dies ist wichtig für die patientenzentrierte Behandlung, aber auch für die Patientensteuerung und verbundene Schonung von klinischen Ressourcen bzw. den Schutz vor Überlastung in den verschiedenen Sektoren, in welchen Notfallversorgung stattfindet. Es sollte durchaus überlegt werden, ob vor diesem Hintergrund der präklinischen Notfallmedizin FachärztInnen mit der neu geschaffenen Zusatzbezeichnung „Klinische Akut- und Notfallmedizin“ einen zentralen Beitrag zur adäquaten Versorgung leisten könnten. Sie dienen aufgrund ihrer Erfahrung aus der unmittelbaren klinischen Akutversorgung ohnehin schon als Bindeglied und Gatekeeper in die Klinik.

### Weiterbildung und Fortbildung im notärztlichen Bereich notärztliche/r KollegIn

Eine einheitliche Pflicht zur notfallmedizinischen Fortbildung gibt es in Deutschland nicht. Die Fortbildungsordnungen der Landesärztekammern basieren strukturell und inhaltlich auf der Muster-Fortbildungsordnung der Bundesärztekammer und sind im Fünften Buch Sozialgesetzbuch (SGB V) verankert [8]. Diese schreibt jedoch nur für FachärztInnen eine kontinuierliche Fortbildung vor, welche nicht spezifisch zu Themen oder Fachbereichen erfolgen muss. Einzelne Bundesländer, wie z. B. Hessen und Bayern, sehen eine verpflichtende Fortbildung im Bereich der Notfallmedizin vor [9].

### Internationaler Vergleich

Rettungsdienstsysteme mit Arztbeteiligung weisen im internationalen Vergleich eine große Heterogenität auf. Teilweise gibt es deutlich weniger Regularien als in Deutschland. Anderseits gibt es Länder, welche die Standards genauer regulieren und eine kontinuierliche Weiterbildung verlangen. Im Vereinigten Königreich ist beispielsweise ein Facharzt in Notfallmedizin, Intensivmedizin oder Anästhesie sowie ein einjähriges strukturiertes Vollzeittraining in Pre-Hospital Emergency Medicine (PHEM) erforderlich, um in der präklinischen Notfallversorgung als NotärztIn tätig zu sein [10]. Bezüglich einer kontinuierlichen Weiterbildung wird z. B. in der Schweiz eine Re-Zertifizierung der Berechtigung zum Führen der Zusatzbezeichnung Notfallmedizin alle 5 Jahre mit dem Nachweis notfallmedizinisch spezifischer Fortbildungen verlangt [11].

Es gibt hier jedoch keine den Autoren bekannten Untersuchungen, die die Auswirkungen dieser Bemühungen hin zu einer kontinuierlichen verpflichtenden Fort- und Weiterbildung in Bezug auf die Qualität der präklinischen notärztlichen Versorgung im Vergleich zu freiwilligen Fortbildungsoptionen umfänglich analysieren.

### Ausbildung der NotfallsanitäterInnen

Die Ausbildung des nichtärztlichen Personals ist mit der Einführung der NotfallsanitäterIn erstmals einheitlich als Ausbildungsberuf in einer dreijährigen Ausbildung geregelt (Notfallsanitätergesetz – NotSanG; [12]). Die Einführung erfolgte 2014 mit Übergangsregelungen für RettungsassistentInnen, die bereits im Rettungsdienst gearbeitet haben. Hierdurch konnten diese mit entsprechenden Fristen und einem verkürzten Curriculum die Prüfung zum/zur NotfallsanitäterIn ablegen.

In dem Gesetz ist wie im ärztlichen Bereich keine kontinuierliche Weiter- und Fortbildung im Sinne einer Erhaltung der Fachkenntnisse verankert.

## Hilfsmittel für die Einschätzung der PatientInnen am Einsatzort

### Die Hilfsmittel zur Ersteinschätzung des Rettungsdienstes

Die apparativen diagnostischen Möglichkeiten in der Präklinik sind im Vergleich zur klinischen Umgebung stark eingeschränkt. Aus diesem Grund ist es wichtig, dass verlässliche Instrumente für die RettungsdienstmitarbeiterInnen oder NotärztInnen zur Verfügung stehen. In erster Linie kommt hier eine strukturierte Erhebung der Anamnese mit verschiedenen Scores zur Einschätzung der Krankheitsschwere zur Anwendung.

Ein weiteres zentrales Instrument ist die körperliche Untersuchung. An apparativer Diagnostik stehen neben dem Stethoskop im Rettungsdienst regelhaft ein Kreislaufmonitoring sowie Blutzuckermessgeräte zur Verfügung.

Aufgrund technischer Entwicklung gibt es vermehrt die Möglichkeiten für die präklinische Laboranalytik. Seit 1990 gibt es bereits Empfehlungen zum präklinischen Einsatz von Point-of-Care-Blutgasanalyse-Geräten, welche jedoch im Alltag noch die Ausnahme darstellen und hauptsächlich im Bereich des Intensivtransportes zu finden sind [13].

Weiterhin besteht die Möglichkeit zur Messung einzelner Werte, wie etwa des hochsensitiven Troponins. Da Symptome aber meist verschiedene klinische Ursachen haben, stellt sich die Frage, ob diese Messung einen wirklichen Nutzen und eine Sicherheit für die PatientInnen bietet. Die Relevanz für die präklinische Notfallmedizin ist derzeit unklar.

Jenseits dieser Analyseverfahren gibt es Initiativen und Projekte, die weitergehende apparative Diagnostik- und Therapieverfahren in die präklinische Versorgung einbinden. Eine flächenhafte Verbreitung besteht hier jedoch nicht. Kürzlich publiziert wurde das Projekt „Mobile präklinische Computertomographien“ im Sinne einer mobilen Stroke Unit zur präklinischen Schlaganfalldiagnostik [14].

Durch die Entwicklungen im Bereich der Ultraschallgeräte kamen in den letzten Jahren immer günstigere portable Ultraschallgeräte auf den Markt. Die präklinische Point-of-Care-Sonographie wird daher vermehrt genutzt. Der Einsatz ist einfach, die Schulung für die KollegInnen überschaubar und der Nutzen für die Versorgung der PatientInnen konnte mittlerweile in Studien belegt werden [15]. So ist zu erwarten, dass es zu einer weiteren Verbreitung in den nächsten Jahren kommen wird.

### Schemata im Rettungsdienst

In den letzten 3 Jahrzehnten hat sich die strukturierte Abarbeitung von Notfällen anhand von Schemata in Akronymform im Rettungsdienst etabliert. Diese Schemata bieten den Vorteil, komplexe Geschehen in Einzelteile zu zerlegen und nach Dringlichkeit standardisiert abzuarbeiten, ohne dabei etwas zu übersehen oder zu vergessen. Es gibt nur vereinzelt wissenschaftliche Arbeiten zum Nutzen dieser Vorgehensweise [16, 17]. Nichtsdestotrotz ist dieses Vorgehen sowohl in notärztlicher als auch in der nichtärztlichen Ausbildung des Rettungsdienstpersonals Standard. Ihren Ursprung finden die Schemata im Advanced Trauma Life Support (ATLS), einem Kurs zur strukturierten Abarbeitung von polytraumatisierten PatientInnen. Das Herzstück des Kurses ist das ABCDE-Schema welches mittlerweile an vielen, auch nichttraumatologischen NotfallpatientInnen angewendet wird.

Darüber hinaus sind für verschiedenste weitere Situationen Akronyme entwickelt worden. Im Folgenden wird eine Auswahl dieser vorgestellt. Es besteht kein Anspruch auf Vollständigkeit, sondern es wurde die Relevanz für Notfallgeschehen herangezogen.

#### SSSS-Schema (Scene, Safety, Situation, Support)

Als erster Schritt beim Eintreffen an der Einsatzstelle erfolgt vor Betreten der Örtlichkeit zunächst eine Beurteilung der Lage mittels des SSSS-Schemas (Tab. 1).

**Table 1:** 

*S* Scene	Einsatzstelle beurteilen
*S* Safety	Eigen- und Fremdgefährdung abschätzen, PSA anlegen
*S* Situation	Verletzungsmechanismus, Unfallhergang, Anzahl PatientInnen abschätzen
*S* Support	Eventuell weitere Einsatzkräfte (Polizei, Feuerwehr) nachfordern

*PSA* persönliche Schutzausrüstung

Hier werden sowohl die Aspekte der Eigensicherung, des Abgleiches der gemeldeten Lage mit der tatsächlichen Lage als auch zusätzlich notwendige Einsatzkräfte zur Verstärkung adressiert.

#### Primary Assessment mittels ABCDE-Schemas

Bei dem/der PatientIn angekommen, erfolgt eine Ersteinschätzung (Primary Assessment) nach dem ABCDE-Schema (Tab. 2). Dieses Schema hat sich inzwischen als ein zentrales Schema für die Evaluation und Versorgung von NotfallpatientInnen etabliert.

**Table 2:** 

	Untersuchungen	Maßnahmen
*A* Airway	Atemweg frei, Atmung vorhanden?	Atemwegssicherung, bei fehlender Atmung CPR
*B* Breathing	Atemfrequenz, Tiefe, Auskultation, SpO2	Sauerstoffgabe, Inhalation, Beatmung
*C* Circulation	Puls, Rekapillarisierungszeit, Blutdruck, Herzfrequenz, EKG, Stauungszeichen	Intravenöser/ossärer Zugang, Volumengabe, Medikamentengabe
*D* Disability	GCS, Pupillen, fokal neurologisches Defizit (FAST- u. BE-FAST-Schema, vgl. Tab. 3 und 4), Blutzucker	Gabe von Glucose
*E* Exposure	Bodycheck, Temperatur, Anamnese, Fremdanamnese	Entkleiden, Wärmeerhalt

*CPR* kardiopulmonale Reanimation; *GCS* Glasgow Coma Score; *SpO2* periphere Sauerstoffsättigung

Dieses Schema ist ubiquitär in der Notfallmedizin verbreitet und anwendbar und wird sowohl in der Ausbildung der NotfallsanitäterInnen als auch den gängigen Reanimationskursen der American Heart Association (AHA), des European Resuscitation Counsil (ERC) sowie den Lehrkonzepten des PHTLS (Prehospital Trauma Life Support) und des ATLS gelehrt. Die Schemata unterscheiden sich leicht je nach Kurssystem und ob der Anwendung je nach Krankheitsbild.

#### *c*ABCDE oder *x*ABCDE

Weiterentwicklung des ABCDE-Schemas sehen die aktuellen ATLS-Leitlinien im Bereich der taktischen Medizin vor. Durch das Voranstellen des *c* (Critical Bleeding, kritische Blutung) bzw. *x* (Exsanguination, Ausblutung) vor das ABCDE versucht man den Blick auf zu erwartende lebensbedrohliche Blutungen zu lenken. Diese gilt es primär zu therapieren [18].

#### FAST und BE-FAST

Zur Beurteilung eines fokal-neurologischen Defizites zur Erkennung eines Schlaganfalls findet das FAST-Schema Anwendung (Tab. 3). Studien zeigen, dass durch dieses Schema 69–90 % der Schlaganfälle des vorderen Stromgebietes erkannt werden, 40 % der Schlaganfälle des hinteren Stromgebietes jedoch verpasst werden [19]. Daher wird in einigen Lehrbüchern das FAST-Schema zum BE-FAST-Schema erweitert (Tab. 4). B für „Balance“ und E für „Eye“ sollen Funktionen der hinteren Strombahn abbilden. In ersten Studien zeigte sich eine Sensitivität zur Erkennung eines Schlaganfalles von 94 % [20]. Diese Daten wurden jedoch im klinischen Bereich erhoben und in präklinischen Studien zeigte sich keine Verbesserung gegenüber dem FAST-Schema [19, 21]. Hier bedarf es weiterer Studien zur Validierung. Allerdings kann das Schema auch aktuell sicher eingesetzt werden und ermöglicht in der präklinischen Versorgung eine gute Einschätzung der PatientInnen.

**Table 3:** 

*F* Face	Faziale Parese
*A* Arm	Hemiparese, Sensibilitätsdefizit
*S* Speech	Dysarthrie
*T* Time	Sofortiger Transport in Stroke Unit

**Table 4:** 

*B* Balance	Gangstörung, Schwindel, Koordinationsstörung
*E* Eyes	Doppelbilder, Sehstörung, Visusverlust
*F* Face	Faziale Parese
*A* Arm	Hemiparese, Sensibilitätsdefizit
*S* Speech	Dysarthrie
*T* Time	Sofortiger Transport in Stroke Unit

#### SAMPLE-Anamnese

Nach der Ersteinschätzung und der Behandlung vitaler Probleme erfolgt im sogenannten Secondary Survey (Zweituntersuchung) eine strukturierte Anamnese. Hierfür hat sich das SAMPLE-Schema etabliert. Mit diesem wird die Anamnese in der präklinischen Versorgung strukturiert. Außerdem gibt es verschiedene Abwandlungen, wie etwa das SAMPLERS-Schema, erweitert um Risikofaktoren und Schwangerschaft (Tab. 5).

**Table 5:** 

*S* Symptoms	Aktuelle Beschwerden
*A* Allergies	Allergien und Unverträglichkeiten
*M* Medication	Aktuelle Medikation
*P* Past Medical History	Vorerkrankungen und vorherige Operationen
*L* Last Meal	Letzte Mahlzeit
*E* Events	Was war vor dem Vorfall, was hat ihn ausgelöst?
*R* Risk Factors	z. B. Alkohol oder Drogenabusus
*S* Schwangerschaft	Könnte eine Schwangerschaft vorliegen oder liegt eine vor

## Steuerung der weiteren Versorgung

Nach der präklinischen Versorgung und Ersteinschätzung werden die NotfallpatientInnen üblicherweise einer weiteren Behandlung zugeführt. Der/die PatientIn muss den Beschwerden und dem klinischen Zustand gemäß eine adäquate Behandlung erhalten. Dies kann eine Vorstellung bei einem/einer niedergelassenen KollegIn bei ambulant behandelbaren Problemen sein. Häufiger kommt es aber zu einer Vorstellung in einer Notaufnahme. Hier ist nochmals zu klären, ob der/die PatientIn eine spezifische Therapie braucht, welche einer Versorgung in einem dafür ausgelegten Zentrum bedarf.

### Ambulante Versorgung im Rettungsdienst

Der Rettungs- und Notarztdienst hat sich im Laufe der letzten Jahre immer mehr weg von der reinen Stabilisierung der Vitalparameter hin zu einer allgemeinen Akutmedizin entwickelt. Das Einsatzspektrum hat sich hierdurch gewandelt. Die rechtliche Grundlage der Versorgung des Rettungsdienstes hat sich aber nicht verändert.

Nach §60 SGB V ist der Rettungsdienst als Transportaufgabe definiert, die streng genommen nur über anfallende Patiententransporte zu finanzieren ist. Eine rein ambulante Versorgung ist hier nicht direkt vorgesehen [22]. Andererseits stellt sich die Frage nach der Patientensicherheit im Rahmen einer ambulanten Behandlung durch den Rettungsdienst. Eine rechtliche Grundlage zur ambulanten Behandlung durch nichtärztliches Rettungsdienstpersonal gibt es derzeit nicht [23]. Aktuell ist eine ärztliche Vorstellung erforderlich, sei es ein/eine hinzugezogene NotärztIn, bei der/dem HausärztIn oder der/dem klinischen NotfallmedizinerIn in der Notaufnahme. Die flächendeckende Einführung einer TelenotärztIn könnte hier weitere Verbesserung und Erleichterung generieren.

Das Sachverständigengutachten zur bedarfsgerechten Steuerung der Gesundheitsversorgung empfiehlt zur Entlastung der klinischen Notfallversorgung dem Rettungsdienst die Option einzuräumen, PatientInnen direkt in einer Praxis vorzustellen. Eine aktuelle Arbeit sieht jedoch nur das Potenzial, die Belastung der Notaufnahmen um 1 % zu reduzieren [24]. Entscheidend ist hier die klinische Erfahrung der Behandelnden in der Präklinik, da eine individuelle Einschätzung jedes Falles erforderlich ist.

In Hessen findet gerade ein Modellversuch statt: „SaN-Projekt: Schnittstellenprojekt zur ambulanten Notfallversorgung“. Hierbei werden Praxen in den landesweiten Bettennachweis IVENA (Interdisziplinärer Versorgungsnachweis) eingepflegt, sodass der Rettungsdienst diese Praxen direkt anfahren kann. Auch findet eine Vernetzung der bundeseinheitlichen Telefonnummer 116.117 des Kassenärztlichen Bereitschaftsdienstes mit der 112 der Leitstelle statt. Weiterhin erfolgt direkt beim Anruf eine Triagierung mittels des Software-Programms *SmED* (Strukturierte medizinische Ersteinschätzung in Deutschland; [25]).

Die Weiterbildung Notfallmedizin legt auf diesen Bereich der ambulanten Versorgung aktuell keinen großen Fokus. Um aber eine wirkungsvolle Entlastung der stationären Versorgungsbereiche zu ermöglichen, bedarf es hier sicher weiterer Bemühungen. Gerade im Bereich der Qualifikation der NotärztInnen sollte nachgeschärft werden. So können umfassende Erfahrungen in der klinischen Notfallmedizin vorteilhaft für die allgemein- oder akutmedizinische Risikostratifizierung sein.

### Planung der stationären Behandlung

Erfordert das Krankheitsbild der PatientInnen eine stationäre Versorgung wird das nächste geeignete Krankenhaus, welches sich an der Notfallversorgung beteiligt, ermittelt und der/die PatientIn dort entweder elektronisch (z. B. mittels IVENA) oder telefonisch angemeldet. Hier zeigt sich aus der Erfahrung der letzten Jahre im Rahmen der COVID-19-Pandemie, dass die Kliniken sich aufgrund von Versorgungsengpässen vermehrt von der Notfallversorgung abmelden. Daher ist bei der Anmeldung auf den Status des Krankenhauses zu achten und ggf. alternativ eine andere Klinik anzufahren. Die optimale Versorgung der PatientInnen darf darunter nicht leiden. Es sollte aber auch die Versorgungsstruktur insgesamt beachtet werden – wenn große Häuser vermehrt PatientInnen mit einfachen Krankheitsbildern versorgen, kann es in der Spezialversorgung in diesen Häusern ebenfalls zu Engpässen kommen. Daher ist es wichtig, dass dem Rettungsdienstpersonal sein Einsatzgebiet und die stationären Versorgungsmöglichkeiten bekannt sind und es mit den Möglichkeiten der Kliniken vertraut ist, um zu einer guten Patientensteuerung beizutragen.

Zur Verbesserung des Schnittstellenmanagements zwischen Klinik und Rettungsdienst gibt es in Deutschland beispielsweise das IVENA und das Rescue-Track-System. Beide Systeme beinhalten einen durch den Rettungsdienst und die Leitstelle einsehbaren Nachweis der freien Betten. Die Systeme beruhen darauf, dass die entsprechenden Klinikabteilungen ihre freien Bettenkapazitäten selbstständig ab- und wieder anmelden.

### Stationäre Versorgung von kritischen Krankheitsbildern

#### Die Schwerverletztenversorgung

Die richtige Einschätzung der Verletzungsschwere mit den möglichen Komplikationen und die folgende Auswahl der Zielklinik stellen die Weichen für die weitere Prognose der verletzten PatientInnen. Das Weißbuch Schwerverletztenversorgung der Deutschen Gesellschaft für Unfallchirurgie (DGU) hat Kriterien zur Aufnahme in den Schockraum eines überregionalen Traumazentrums aufgestellt [26]. Gemäß DGU sollten PatientInnen innerhalb einer Transportzeit von 30 min in ein regionales oder überregionales Traumazentrum transportiert werden können. Ist diese Zeitvorgabe nicht einzuhalten, ist die nächstgelegene Einrichtung zur Basisversorgung anzusteuern [26].

Die Versorgung von Brandverletzten sollte zunächst in der Notaufnahme des nächstgelegenen geeigneten Krankenhauses erfolgen, da etwaige schwerwiegende Begleitverletzungen vorrangig behandelt werden müssen. Wird eine Verlegung in ein Zentrum angestrebt, ist hier eine luftgebundene Verlegung aufgrund der Distanzen und des schonenden Transportes häufig sinnvoll.

#### Herz-Kreislauf-Stillstand

Seit den Leitlinien des Deutschen Rats für Wiederbelebung/German Resuscitation Council (GRC) von 2015 wird die weitere Versorgung von PatientInnen mit außerklinischem Herz-Kreislauf-Stillstand in einem Cardiac-Arrest-Center gefordert. Eine interdisziplinäre Arbeitsgruppe hat in einem Update 2021 die entsprechenden strukturellen Voraussetzungen und Qualitätsindikatoren für diese Zentren definiert [27, 28].

Diese sollen eine standardisierte und hochwertige an die aktuellen Leitlinien angepasste Therapie von reanimierten PatientInnen gewährleisten. Ist ein solches Zentrum in annehmbarer Zeit erreichbar, sollten reanimierte PatientInnen dort vorgestellt werden. Dies kann zu einem besseren Outcome für die PatientInnen führen [29].

#### Schlaganfallversorgung

Die Versorgung des akuten Schlaganfalls ist aufgrund eines engen Therapiefensters zeitkritisch und soll in zertifizierten Stroke Units erfolgen.

Einen Paradigmenwechsel erfuhr die Schlaganfallversorgung im Jahr 2015, nachdem die Deutsche Gesellschaft für Neurologie (DGN) in der Ergänzung ihrer S2K-Leitlinie „Akuttherapie des ischämischen Schlaganfalls“ aufgrund überzeugender Studiendaten die mechanische Thrombektomie bei großem Gefäßverschluss (Large Vessel Occlusion) empfahl [30]. Da die mechanische Thrombektomie nur in spezialisierten neurovaskulären Zentren angeboten werden kann, steht der Rettungsdienst nun vor der Herausforderung möglichst jene PatientInnen zu identifizieren, welche von einer solchen Therapie profitieren. Solche, die von dieser Intervention nicht profitieren, sollten in die nächstgelegene Stroke Unit transportiert werden. Hier gibt es noch kein validiertes System zur guten Differenzierung, daher ist die Unterscheidung aktuell schwierig [31].

## Der Massenanfall von Verletzten (MANV), Großschadensereignis und die Katastrophe

Eine andere Situation bezüglich der Beurteilung der PatientInnen ergibt sich im Falle eines Massenanfalls von Verletzten (MANV) oder bei einer katastrophenmedizinischen Situation. Hier übersteigt die Anzahl der PatientInnen die vorhandenen Ressourcen kurzfristig oder auch längerfristig.

In einer solchen Situation muss die Herangehensweise des Rettungsdienstes umgestellt werden, weg von der individuellen Versorgung jedes/jeder PatientIn mit allen vorhandenen Ressourcen hin zu einer Schonung und dem gezielten Einsatz vorhandener Ressourcen in der präklinischen Versorgung. Auch die Ressourcen der stationären Versorgung werden durch die gezielte Vorstellung von PatientInnen geschont.

Ein zentrales Tool zur Evaluation und Einschätzung der Situation ist die Sichtung. Es gilt, eine schnelle Ersteinschätzung für alle PatientInnen zu erhalten und den Ressourcenbedarf abzuschätzen.

### Definitionen

#### MANV oder MANI

„Der Massenanfall von Verletzten (MANV) oder Erkrankten/Infizierten (MANI) ist ein Notfall mit einer größeren Anzahl von Verletzten oder Erkrankten sowie anderen Geschädigten oder Betroffenen, der mit der vorhandenen und einsetzbaren Vorhaltung des Rettungsdienstes aus dem Rettungsdienstbereich versorgt werden kann“ [32].

#### Großschadensfall

„Ein Ereignis mit einer so großen Anzahl von Verletzten oder Erkrankten sowie anderen Geschädigten oder Betroffenen, dass es mit der vorhandenen und einsetzbaren Vorhaltung des Rettungsdienstes aus dem Rettungsdienstbereich nicht bewältigt werden kann“ [32].

#### Katastrophe

„Ein über das Großschadensereignis hinausgehendes Ereignis mit einer wesentlichen Zerstörung oder Schädigung der örtlichen Infrastruktur, speziell der medizinischen Versorgungseinrichtungen. Es kann im Rahmen der medizinischen Versorgung mit den Mitteln und Einsatzstrukturen des Rettungsdienstes alleine nicht bewältigt werden“ [32].

### Die Abarbeitung des MANV

Die Abarbeitung eines MANV ist nicht bundeseinheitlich geregelt und kann regional von der folgenden Beschreibung abweichen. Die spezifischen Begrifflichkeiten werden neben anderen Begrifflichkeiten des Rettungsdienstes im Normblatt DIN 13050:2002-09 definiert. Abläufe und Vorgehensweisen werden hier nicht beschrieben.

#### Organisatorischer Ablauf

Die ersteintreffende NotärztIn führt die Sichtung durch und ist organisatorische ärztliche Leitung des Einsatzes bis zum Eintreffen des/der vom Landkreis bestimmten Leitende NotärztIn (LNA). Mit dem/der organisatorischen Leiter Rettungsdienst (OrgL) bildet der/die LNA die Einsatzleitung des Rettungsdienstes. Diese ist je nach Bundesland der Feuerwehr unterstellt ist [33].

Zentrale Aufgabe sind die Sichtung der Verletzten sowie die Festlegung der Behandlungs- und Transportkapazität, um einer möglichst großen Zahl von PatientInnen das Überleben zu sichern. Dafür gibt es verschiedene Algorithmen-basierte Systeme, welche alle auf den sogenannten Sichtungskategorien beruhen (Tab. 6).

**Table 6:** 

Sichtungskategorie (SK)	Beschreibung	Konsequenz
SK I (rot)	Vital bedroht	Sofortbehandlung
SK II (gelb)	Schwer verletzt/erkrankt	Dringliche Behandlung
SK III (grün)	Leicht verletzt/erkrankt	Nicht dringliche Behandlung
SK IV (blau)	Ohne Überlebenschance	Palliative Versorgung
EX (schwarz)	Tot	Vorläufige Todesfeststellung

Gängige Systeme sind PRIOR (Primäres Ranking zur Initialen Orientierung im Rettungsdienst), mSTaRT (Modified Simple Triage and Rapid Treatment), FTS (Field Triage Score) und ASAV (Amberg-Schwandorf-Algorithmus für die Vorsichtung; [34]).

#### Ablauf der Sichtung

Gemäß den Empfehlungen des Bundesamtes für Katastrophenschutz (BBK) erfolgt zunächst eine Ersteinschätzung der Lage [35]. Dem SSSS-Schema folgend sollte sich ein grober Überblick verschafft, nach Gefahren und grober Patientenzahl geschaut und die Lage „auf Sicht“ an die Leitstelle zurückgemeldet werden, sodass diese frühzeitig weitere Einsatzkräfte alarmieren kann.

Ein besonderes Augenmerk muss auf den Charakter der Lage und damit einhergehende Aspekte der Eigensicherung gelegt werden. So ist zwischen einer Feuerwehrlage, einer Polizeilage und einer Rettungsdienstlage zu unterscheiden. Bei Eintreffen des Rettungsdienstes kann sich das Geschehen noch entwickeln. Daher wird zwischen einer dynamischen und einer statischen Lage unterschieden. Wichtig ist, dass sich der Rettungsdienst niemals in die direkte Gefahrenzone begibt und je nach Einsatzlage auf Freigabe der Feuerwehr oder Polizei wartet oder Bereiche zur Übergabe von PatientInnen definiert. Das Vorgehen und die genauen Abläufe sind im Detail für verschiedene Rettungsdienstbereiche entsprechend unterschiedlich.

Im nächsten Schritt folgt die Vorsichtung [33]. Im Rahmen der Vorsichtung werden die Verletzten nacheinander basierend auf Algorithmen einzeln betrachtet. Dabei erfolgen eine vorläufige, standardisierte medizinische Zustandsbeurteilung sowie eine rudimentäre Behandlung lebensbedrohlicher Verletzungen. Vorrangiges Ziel der Vorsichtung ist es, möglichst schnell die „roten PatientInnen“ (Sichtungskategorie I, vital bedroht, Tab. 6) zu identifizieren, die als Erstes behandelt und transportiert werden müssen [35].

Die Kategorien folgen einem Ampelsystem, die Beurteilung wird mittels Ansteckkarten für die jeweiligen PatientInnen ausgewiesen, sodass für nachrückende Kräfte der Status erkennbar ist.

Aufgrund der Dynamik kann eine regelmäßige Reevaluation der Sichtungskategorien nötig werden. Im weiteren Versorgungsverlauf bedarf es dafür immer einer ärztlichen Sichtung der PatientInnen.

### Rechtliche Aspekte der Sichtung

Die Sichtung eines MANV hat eine besondere ethische und rechtliche Situation. Dies ist der Tatsache geschuldet, dass die Sichtung nicht wie in einer sonstigen notfallmedizinischen Situation dahin gehend geführt wird, die am schwersten Verletzten am schnellsten zu behandeln. Vielmehr wird versucht, durch eine Sichtung eine optimale Ressourcenallokation zu erreichen. Dies kann aber auch bedeuten, dass PatientInnen mit der niedrigsten Überlebenswahrscheinlichkeit keine Behandlung erfahren oder erst verzögert behandelt werden. Durch diese Vorgehensweise soll eine möglichst hohe Anzahl an PatientInnen gerettet werden.

Die Sichtung ist weder in den Katastrophenschutzgesetzen des Bundes noch in denen der Länder konkret geregelt [36]. Die rechtliche Beurteilung beruft sich daher auf das Grundgesetz (GG), den etwaigen Verstoß gegen die Garantie der Menschenwürde (Art. 1 I GG) und das Recht auf Leben und körperliche Unversehrtheit (Art. 2 II 1 GG; [32]). Nach gängiger Meinung gilt ein Behandlungsanspruch des Einzelnen nur im Sinne einer Mindestversorgung und Rationierungsmaßnahmen im Sinne einer Ungleichbehandlung sind zulässig. Die entscheidende Frage ist, welche Kriterien zulässig sind. Es gilt hier, die Kriterien der Dringlichkeit, Erfolgsaussicht und des Aufwands der medizinischen Behandlung in Bezug auf die Maximierung der Zahl an Überlebenden in ein optimales Verhältnis zueinander zu bringen [32].

## Fazit

Die präklinische Einschätzung der PatientInnen kann durch Standards und Akronyme erleichtert und standardisiert werden. Die technischen Entwicklungen geben dem Rettungsdienstpersonal und NotärztInnen neue Möglichkeiten der Diagnostik. Entscheidend bleibt aber die Erfahrung der NotfallsanitäterInnen oder NotärztInnen, um eine gute Einschätzung der PatientInnen zu treffen. Hier kommen durch die gesellschaftlichen Entwicklungen neue Herausforderungen auf das Personal zu. Es gilt, durch Aus‑, Weiter- und Fortbildung die Voraussetzung für die Bewältigung zu schaffen. Weiterhin bedarf es auch der Weiterentwicklung von innovativen Konzepten (z. B. TelenotärztInnen), klinischer Erfahrung in ambulanter Behandlung und Verweis ins ambulante System, um begrenzte klinische Ressourcen zu schonen.
